# *In Vitro* and *in Vivo* Models of Non-Alcoholic Fatty Liver Disease (NAFLD)

**DOI:** 10.3390/ijms140611963

**Published:** 2013-06-05

**Authors:** Giridhar Kanuri, Ina Bergheim

**Affiliations:** Department of Nutritional Sciences, SD Model Systems of Molecular Nutrition, Friedrich-Schiller-University, Dornburger Str. 25-29, D-07743 Jena, Germany; E-Mail: giridhar.kanuri@uni-jena.de

**Keywords:** animal models, *in vitro* models, non-alcoholic fatty liver disease, insulin resistance, diet

## Abstract

By now, non-alcoholic fatty liver disease (NAFLD) is considered to be among the most common liver diseases world-wide. NAFLD encompasses a broad spectrum of pathological conditions ranging from simple steatosis to steatohepatitis, fibrosis and finally even cirrhosis; however, only a minority of patients progress to end-stages of the disease, and the course of the disease progression to the later stages seems to be slow, developing progressively over several years. Key risk factors including overweight, insulin resistance, a sedentary life-style and an altered dietary pattern, as well as genetic factors and disturbances of the intestinal barrier function have been identified in recent years. Despite intense research efforts that lead to the identification of these risk factors, knowledge about disease initiation and molecular mechanisms involved in progression is still limited. This review summarizes diet-induced and genetic animal models, as well as cell culture models commonly used in recent years to add to the understanding of the mechanisms involved in NAFLD, also referring to their advantages and disadvantages.

## 1. Introduction

Throughout the last decades the prevalence of overweight and obesity has increased dramatically worldwide. Overweight and obesity have been identified to be key risk factors for many chronic diseases including cardiovascular diseases, type 2 diabetes and lipid disorders, but also non-alcoholic fatty liver disease (NAFLD) (for review see [1]). Indeed, NAFLD is now thought of as the hepatic manifestation of the metabolic syndrome, and is by now regarded as one of the most common liver diseases worldwide [2]. It is estimated that about 20% of the general adult population of most Westernized countries have hepatic steatosis and that ~2%–3% of adults even suffer from non-alcoholic steatohepatitis (NASH) [3].

**Figure 1:**
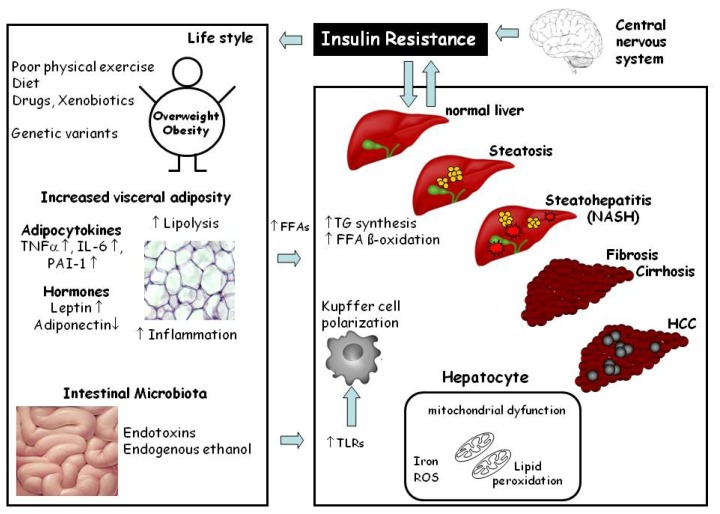
Schematic drawing of the molecular mechanisms involved in the development and progression of non-alcoholic fatty liver disease (NAFLD). The risk factors such as being overweight, visceral adiposity, adipocytokines may increase the flow of free fatty acids (FFAs) to the liver. Alterations of intestinal microbiota and increased permeation of bacterial endotoxins, from the gut may activate Toll-like receptor signaling cascades and lead to a M1 polarization of macrophages (e.g., of Kupffer cells and infiltrating macrophages). All these events (e.g., increased FFAs, adipocytokines, endotoxins, insulin resistance, macrophage polarization) may lead to the development of NAFLD. (Modified from Krawczyk *et al.* [4]).

The earliest and most common type of NAFLD is simple steatosis, which has long been thought to be a relatively benign state of liver injury; however, results of human studies indicate that fatty livers are more vulnerable to injury from various causes [2] and progress more rapidly to steatohepatitis, increasing the probability of further liver related morbidity and mortality [5]. Despite intense research efforts, molecular mechanisms involved in the onset, but also the progression of the disease, are still not fully understood. Indeed, in recent years it has been proposed that NAFLD may actually result as a consequence of multiple hits among which gut-, as well as adipose tissue-derived, factors may play a central role (for overview see [6] and Figure 1). Accordingly, universally accepted therapeutic options other than lifestyle modification including weight reduction diets and exercise are not yet available.

Due to ethical limitations in regards to tissue collection, but also therapeutic interventions (e.g., testing of drugs), and as the occurrence even of steatosis, but more so the progression to later stages of the disease (e.g., NASH, fibrosis or cirrhosis), may require a long period of time to study, animal models resembling conditions of the early phases of NAFLD in humans (e.g., steatosis and steatohepatitis) have been found to be useful tools to investigate mechanisms and pathophysiology underlying the development of NAFLD. The current review will focus primarily on dietary and genetic rodent animal models of NAFLD, and also on *in vitro* models and some ‘non-rodent’ animal models commonly used to study molecular mechanisms involved in the NAFLD.

## 2. Histopathology and Pathogenesis of NAFLD

The earliest stage of NAFLD is hepatic steatosis characterized by the deposition of cytoplasmic triglycerides as macro- and/or microvesicular lipid vacuoles in more than 5% of hepatocytes (for overview see [7] and Figure 1). The excessive accumulation of triglycerides in the hepatocytes arises from a dysbalance of triglyceride acquisition and removal, which seems primarily to result from (i) a hypercaloric and/or unbalanced diet, (ii) an increased de novo synthesis of triglycerides or (iii) enhanced lipolysis in adipose tissue (for overview also see [7]). Hepatic steatosis is often self-limited; however, it can progress to NASH distinguished from simple steatosis by the presence of hepatocellular injury, inflammatory infiltrate and/or collagen deposition (e.g., fibrosis) [7]. Fibrosis usually originates in the perisinusoidal regions of zone 3 and may also be present in the periportal area (for overview see [8,9]). Up to now, it is not clear what causes the progression of steatosis to NASH or if steatosis and NASH are distinct disorders (for overview see [6,7]).

## 3. Animal Models of NAFLD

In most patients the development of NAFLD takes years and results from an interplay of several risk factors like overnutrition and/or an inappropriate dietary pattern (e.g., high fat and/or high sugar intake) as well as inadequate energy expenditure due to a sedentary lifestyle and probably genetic susceptibility, all leading to multiple molecular alterations in the human organism (for overview see [6] and Figure 1). Accordingly, animal models used to study the onset of, but also progression of NAFLD to later stages of the disease like NASH or even fibrosis and cirrhosis, should incorporate the following criteria: (i) the pathological patterns and histological alterations found in the different stages of the disease in humans and (ii) the general physiological alterations associated with the disease development in humans (e.g., weight gain, insulin resistance but also impaired intestinal barrier function and adipocytokine release from adipose tissue). Inadequateness in resembling either the liver pathology or the physiological alterations in experimental models of NAFLD will make it difficult to translate results found in such laboratory model systems to the clinical situation and subsequently the development of therapeutic or prevention strategies of the disease. Specifically, an appropriate animal model of NAFLD should display not only steatosis, but also inflammation, liver cell injury (e.g., ballooning of hepatocytes) and should, if long enough extended, also progress to fibrosis. Furthermore, the model should also display metabolic abnormalities like overweight, insulin resistance, impaired glucose tolerance, dyslipidemia and altered adipocytokine profiles, as well as the increased bacterial endotoxin levels frequently found in patients with NAFLD (for overview see [6]).

However, as human behaviour and biology are rather complex, it should be kept in mind when selecting an animal model to study NAFLD, and also when interpreting that data obtained in these models that other factors like physical activity, social environment, psychological stress factors and genetics may also be important contributors to the development of NAFLD in humans.

## 4. Genetic Rodent Models of NAFLD

### 4.1. Ob/ob, db/db and Obese (fa/fa) Zucker Rat

**Figure 2:**
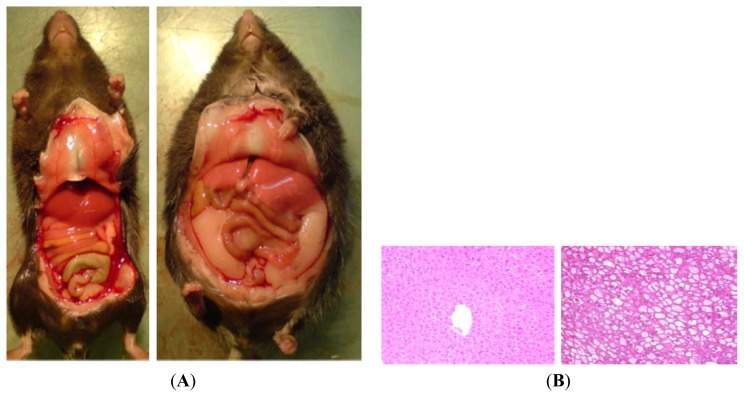
(**A**) Representative photographs of a lean (C57BL/6) and an ob/ob mouse, as well as (**B**) representative photomicrographs of liver sections stained with hematoxylin and eosin (200×).

Among the most frequently used genetic models to study NAFLD are those that exhibit alterations in feeding behaviour (e.g., hyperphagia), which frequently results from defects in the leptin signalling pathways. Indeed, the so-called ob/ob mouse carries a spontaneous mutation in the leptin gene, which results in a leptin deficiency, subsequently leading to a hyperphagic, inactive, obese, and diabetic phenotype accompanied by the development of NAFLD ([10,11] and Figure 2). This model has been extensively studied, and is used as a model for various metabolic diseases, among them NAFLD, despite the fact that the “*ob*” mutation is rare in humans [12–14]. Furthermore, results of association studies of leptin levels with the development of NAFLD are somewhat contradictory, as in older studies leptin levels correlate poorly with the development of NASH, whereas in more recent years, a positive association was found [12–14].

Furthermore, more recent studies also reported an association of a single nucleotide polymorphism on the gene encoding for the leptin receptor with the development of NAFLD [15,16]. However, also unlike the human situation, *ob/ob* mice do not spontaneously progress from steatosis to steatohepatitis but rather require some kind of “second hit” (e.g., a dietary intervention such as a methionine- and choline-deficient (MCD) diet, or a high-fat diet (HFD), or a challenge with small doses of endotoxin) [17,18].

Contrary to what is seen in ob/ob mice, db/db, mice have a natural mutation in the leptin receptor (*ob-Rb*) gene; however, in line with what is found in *ob/ob* mice, *db/db* animals also only develop obesity, insulin resistance, and macrovesicular steatosis and require a “second hit” to progress from steatosis to steatohepatitis [19]. Similar to the latter, the obese (*fa/fa*) Zucker rat also exhibits hyperphagia resulting in hyperinsulinemia, hyperlipidemia and the development of liver steatosis due to a loss of the leptin receptor [20,21].

### 4.2. Agouti Gene [KK-Ay/a]

The heterozygous mutation of the agouti gene (*KK-Ay/a*) has been shown to result in a loss of melanocortin, and an obese phenotype resulting from hyperphagia caused by an impaired hypothalamic appetite suppression, in addition to changes in color. Similar to what is found in ob/ob and db/db mice, this strain of mice develops obesity, insulin resistance, and steatosis in the liver but also does not progress to later stages of the disease. In order to progress to NASH, a “second hit” (e.g., a specific diet, like a MCD diet, a HFD, or a challenge with small doses of endotoxin) is again necessary [22], thereby only partly mimicking the situation found in humans with NAFLD.

### 4.3. Melanocortin 4 Receptor (MC4R)

In recent years, studies have revealed that the melanocortin 4 receptor (*MC4R*) may be a critical factor in the regulation of food intake and body weight [23]. Indeed, several pathogenic mutations of the *MC4R* were identified at a high frequency in severe early-onset obesity in humans [24]. Mice with targeted disruption of the *MC4R* have been shown to develop late-onset obesity with hyperphagia, hyperinsulinemia, and hyperglycaemia [25,26]. Furthermore, MC4R-KO mice fed a HFD have been shown to exhibit massive hepatic steatosis and altered gene expression of genes related to lipid metabolism [27]. Recently, Itoh *et al*. reported that MC4R-KO mice fed a HFD for up to a year progress from early steatosis through NASH to even liver fibrosis and hepatocellular carcinoma, displaying many metabolic abnormalities also reported in humans with NAFLD (e.g., excessive weight gain, altered blood glucose levels, hyperlipidemia) [28]; however, if alterations of the intestinal barrier function are also present in this model has to our knowledge not yet been studied.

### 4.4. Sterol Regulator Element-Binding Protein 1c (SREBP)

Targeted overexpression of the insulin-regulated transcription factor sterol regulator element-binding protein 1c (*SREBP1*) in adipose tissue has also been shown to results in the development of NAFLD in mice [29,30]. In this model the development of fatty liver was associated with alterations in the differentiation of white adipose tissue and hyperinsulinemia but also mild hepatic inflammation and fibrosis; however, body weight did not differ from wild-types and development of adipose tissue was markedly altered [29].

## 5. Dietary Rodent Models of NAFLD

### 5.1. Methionine- and Choline-Deficient (MCD) Diet

Dietary models to induce NAFLD have relevance to human disease since alterations of dietary pattern and over-nutrition both are being associated with the development of overweight and insulin resistance have been identified to be key risk factors for the development of the disease. However, conversely to what is found in most patients with NAFLD, one of the most frequently used dietary models for NASH, the MCD diet, is a nutrient-deficient dietary model. The MCD diet normally contains substantial amounts of sucrose (e.g., 40%) and low amounts of fat [10%] but is deficient in methionine and choline, both being essential factors in human and animal nutrition. Rodents fed a MCD diet rapidly (e.g., within 1 to 2 weeks) develop hepatic steatosis due to enhanced uptake of fatty acids and decreased secretion of very-low-density lipoproteins from the liver (for overview see [31]). After two weeks, the development of steatosis is followed by necrosis and inflammation and then even progresses to pericellular and pericentral fibrosis. In addition, oxidative stress [32], activation of Toll-like receptor *(TLR*) *4*-dependent signalling cascades in the liver [33] and changes in cytokines as well as adipocytokines occur [33], all contributing to liver injury in this dietary model. Despite the fact that under the MCD diet rodents develop significantly faster and more pronounced liver damage, this dietary feeding regiment has some marked limitations. For instance, the main risk factors for the development of NAFLD found in humans (e.g., insulin resistance and overweight) are lacking; rather, severe weight loss of up to 35% over a period of 4 weeks has been reported [34]. Furthermore, it has been shown in several studies that the responsiveness of different mouse strains towards the MCD diet induced NAFLD varies considerably. Indeed, release of transaminases in different mouse strains can be ranked as follows: A/J > C57BL/6 > C3H/HeN = Balb/c = DBA/2J, whereas long term feeding of an only methionine deficient diet causes a more pronounced liver injury and even hepatocarcinogenesis in DBA/2J than in C57BL/6 mice [35–37].

### 5.2. High Fat Diet (HFD)

Epidemiological studies suggest that a diet rich in fat might be a risk factor for the development of obesity and insulin resistance [38]. Furthermore, fat is the most energy dense macronutrient in human nutrition thereby increasing the odds to develop obesity when consumed in excessive amounts. Accordingly, diets rich in fat (HFD), e.g., 30%–75% of total calories derived from saturated fatty acids (± unsaturated fatty acids) have been proposed to be a useful tool to induced metabolic alterations and NAFLD. Indeed, depending on the duration of the feeding time and combination of fatty acids, rodents fed an HFD display obesity, impaired glucose tolerance, dyslipidemia, increased expression of regulators of lipogenesis (e.g., *SREBP1c* and liver X receptor) and expression of proinflammatory cytokines (for overview see [39,40]). Furthermore, signs of oxidative stress and of increased sensitivity to endotoxin as well as alterations in the Toll-like receptor 4 signaling cascade in the liver and a protection of animals depleted of Kupffer cells against HFD-induced NAFLD suggest that an impaired function of the intestinal barrier is also associated with the development of HFD-induced NAFLD in mice [41]; however, feeding a HFD does not produce as severe degrees of liver injury as those found in the MCD diet models and feeding times necessary to achieve more severe damage are markedly longer than those found for the MCD diet [42,43]. Nevertheless, these diets more closely resemble the pathological and molecular alterations found in humans with NAFLD. It is also for this reason that in recent years HFDs are commonly combined with genetic animal models of NAFLD (e.g., ob/ob mice) [44]. In other reports, the composition of the diet was altered to achieve steatohepatitis (e.g., HFD with lower methionine and choline content) or the application rout of the diet was altered (e.g., ad libitum feeding vs. intragastric force feeding) [45,46].

Indeed, Deng *et al.* [46] introduced an intragastric feeding model adapted from the so called Tsukamoto & French model used in alcoholic liver research [47] to induce steatohepatitis. In this model C57BL/6 mice were fed a high fat diet for 9 weeks intragastically resulting not only in steatohepatitis with beginning fibrosis but also overweight, increased visceral fat, impaired glucose tolerance and increased expression of adipocytokines in white adipose tissue; however, this model requires a surgical procedures and a high maintenance of the animals as the diet is administered through a pump-driven tubing system implanted in the stomach of the animals.

It seems that not only the amount of dietary fat, but also the quality of fat and the fatty acid composition, is important for inducing NAFLD, and maybe also the progression of the disease. A diet containing more unsaturated fat such as fish oil has been shown to result in higher levels of lipid peroxidation and a more pronounced induction of the expression of chemokines and cytokines in the liver than corn oil [48]. Tipoe *et al.* [49] developed a rat model for NASH by feeding a diet that contains 30% unsaturated fat derived from fish oil. Interestingly, after 8 weeks, these animals did not gain more weight than the controls; however, they developed a broad spectrum of features typical for NASH that include elevated ALT levels, increased collagen deposition, and increased fat incorporation, as well as signs of inflammation and necrosis. These results are in line with those of earlier studies that showed that in settings of alcoholic liver damage, an addition of fish oil to the diet as a source of unsaturated fatty acids enhanced liver damage which was associated with marked induction of *CYP2E1* mRNA expression in the liver in comparison to rats fed a diet containing saturated fatty acids. In these studies a significant correlation was found between expression level of *CYP2E1* mRNA and markers of lipid peroxidation (e.g., 4-hydoxynonenal protein adducts in liver tissue) [50,51]. These studies suggest that the harmful effects of unsaturated fatty acids might stem from an enhanced *CYP2E1* mediated metabolism of the fatty acid associated with an enhanced formation of reactive oxygen species [50,51]. Chen *et al.* [52] found that feeding rats a diet fortified with fish oil (205g fish oil + 30 g maize oil/kg) for 6 weeks resulted in a marked mRNA induction of several cytochrom P450 enzymes in the liver; however, activity of superoxide dismutase (SOD) and ethoxyresorufin *O*-deethylase (EROD) was also found to be increased in livers of these animals. In a more recent study by Zong *et al.* [53] it was shown that *CYP2E1* knockout mice fed a high fat diet for 12 weeks were markedly protected from insulin resistance and showed reduced fat accumulation in the liver. Furthermore, van den Berg *et al*. [54] showed that in C57BL/6 mice fed a HFD rich in lard has a greater impact in hepatic insulin sensitivity than a palm oil rich HFD, further suggesting that fatty acid composition may be a critical factor that should also be taken into consideration when choosing a dietary model of NAFLD. In addition, results of several studies suggest that not every mouse strain is similarly responsive to a high fat diet. Indeed, it was shown that both C57BL/6 and 129/SVJ develop NASH and may even display fibrotic alterations in the liver when exposed to a high fat diet for an extended time whereas for A/J mice a similar effect of a high fat diet was not found [55,56]. Furthermore, dietary models combining a high fat diet with cholesterol (1.25%) and even cholate (0.5%) (e.g., as found in the so called Paigen diet) have been shown to induce mild hepatic fibrosis and oxidative hepatocellular damage in rats but also mice within 4 weeks of feeding [57–59]. Table 1 summarizes the composition of the Paigen diet. Taken together, these models clearly demonstrate that not simply high amounts of fat in the diet are causing liver damage but that the type of fat as well as overall composition of the diet in regards to metabolism of dietary fat (e.g., additions like cholate) and the mouse strain may also be important.

### 5.3. Fructose-Rich Diets

Results of several epidemiological and some small clinical studies conducted in different countries (e.g., US, Japan, Israel and Germany) suggest that a shift in dietary patterns towards a sugar rich diet may also be risk factor for the development of NAFLD in humans (for overview see [60,61]). Diets with elevated carbohydrate content (e.g., high-sucrose or high-fructose diets) have also been used to induce the development of NAFLD in mouse models. Studies giving C57BL/6 mice ad libitum access to different mono- and disaccharides in drinking water revealed that fructose had the most damaging effect on the liver despite having the least impact on body weight gain [62]. Furthermore, it was shown that fructose in chow (e.g., up to 60% of total calories derived from fructose) or drinking solutions (e.g., 30% fructose solution) not only leads to the development of NAFLD but also insulin resistance [62–64]. Similar to what was reported from HFD models, diets rich in fructose can lead to oxidative stress, expression of proinflammatory cytokines, and SREBP1c in the liver and elevated endotoxin levels in portal blood as well as alterations of adipocytokine expression in visceral adipose tissue in a dose- and time of- exposure–related manner [62–65]; however, similar to the results found for feeding HFDs, high fructose diets did not cause liver injury as severe as that found in the MCD diet models, despite more closely resembling not only the pathological and molecular alterations but also the dietary patterns found in humans with NAFLD.

### 5.4. Western-Style or Fast Food Diet: Combination of Fat and Sugar

**Figure 3:**
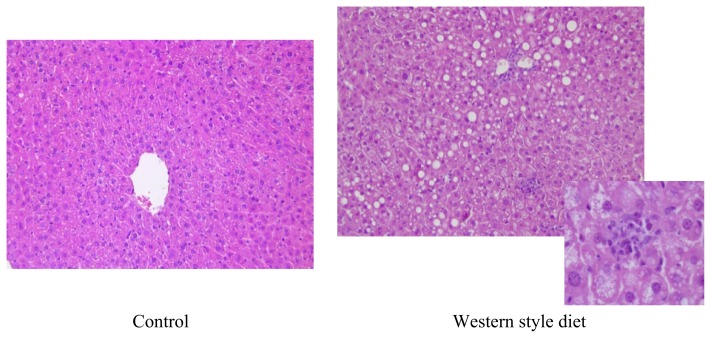
Effect of feeding a Western-style diet on the liver. Representative photomicrographs of hematoxylin and eosin staining of liver sections (200×) of mice fed with control or Western-style diet for 6 weeks.

In analogy to what is found in many Westernized countries in regards to “out-of home dining” or “fast food” rich diets, shown to be risk factor for the development of obesity [66,67] a combination of both fat and fructose -sometimes with slightly elevated cholesterol contents- referred to as Western-style or fast food diet has also repeatedly been used as a dietary model to induce NAFLD in rodents. Charlton *et al*. [68] showed that after feeding C57BL/6 mice for 6 months a fast food diet consisting of a high-fat chow (40% of energy from fat with 2% cholesterol) and drinking water enriched with high fructose corn syrup (HFCS, 42 g/L final concentration) mice not only became overweight and insulin resistant but also developed NASH and showed a gene expression signature of increased fibrosis. In this study, it was also shown that mice fed the fast food diet developed endoplasmatic reticulum stress and lipoapoptosis, both also found in humans with NAFLD. In line with these findings, in our own experiments we found that feeding a liquid diet based Western-style diet in C57/BL6 mice for 6 weeks results in the development of massive hepatic steatosis with marked inflammatory alterations but also increased bacterial endotoxin levels in portal plasma and slightly elevated fasting glucose levels (dietary composition see Table 2 and Figure 3). Furthermore, it was reported by Tsuchiya *et al*. [69] that feeding a fat- and fructose-rich diet for up to 16 weeks leads to hepatic iron overload and increased lipid peroxidation. Somewhat contrary to the results found for animal models only feeding a fructose or fat rich diet, the available data on using a Western-style or fast food diets suggests that animals progress to later stages of NAFLD (e.g., NASH with beginning fibrosis) quicker when being fed these types of diet than when being fed only fat or fructose ad libitium and even seem to gain more weight.

### 5.5. “Non-rodent” Animal Models of NAFLD

Studies have also proposed several non-rodents (like Caenorhabditis elegans, opossum, Ossabaw pig, primates) as models to investigate the pathogenesis of NAFLD [70–72]. The nematode, Caenorhabditis elegans has been employed as a model organism to study obesity and the metabolic syndrome due to the conservation of the pathways that regulate energy and lipid metabolism [70]. Indeed, the insulin like signaling pathway was extensively studied in *C. elegans* [73] and has been proposed to be analogues to mammals; however, as this model lacks a liver clinical application of findings derived in this model organism remain to be determined. Recently studies have suggested that diet rich in cholesterol and fat results in an enormous increase in hepatic total cholesterol levels in *ABCB 4* mutant opossums in comparison to controls when being fed for 24 weeks [74]. Indeed, results of Puri *et al.* [75] suggest that the hepatic cholesterol content in NASH patients was higher in comparison to NAFLD patients without NASH. Hence, opossums might be a novel animal model of NASH resembling the human NASH phenotype; however, further studies are necessary to characterize this animal model. In recent years, studies have also shown that minipigs, like the Ossabaw pigs, may be an excellent model to investigate the metabolic syndrome and type-2 diabetes [76]. In fact, Lee *et al.* [72] have shown that feeding Ossabaw pigs an atherogeneic diet for 24 weeks results in a significantly increased weight gain, and also the development of micro and macrovesicular steatosis and pericellular/perisinusoidal fibrosis when compared to controls; however, large animal models like Ossabaw pigs have the disadvantage to be rather expensive though they resemble human NAFLD more closely than rodents.

### 5.6. *In Vitro* Models for NAFLD

The molecular mechanisms and signalling cascades involved in the progression of NAFLD were first obtained through animal models (e.g., genetic models, diet-induced models) and confirmed through clinical studies (for overview see [77]). However, recent studies have employed *in vitro* approaches to elucidate the molecular mechanisms involved in the progression of the disease. Paradoxically, there is only few data obtained in NAFLD using these models, whereas for other liver diseases *in vitro* models are important tools in research. In general, primary cell cultures and immortalized cell lines are widely used to develop *in vitro* models for research. Primary human hepatocytes [78], Kupffer cells, stellate cells or sinusoidal endothelial cells seems to be physiologically relevant model for clinical conditions [79]; however, ethical issues and limited number of human liver samples make it extremely complicated to use primary human cell cultures. The other option would be primary rodent cells, which depending on the model used may more or less mimic the situation found in humans; however, methods used for isolation need to be well established in order to achieve reproducible results and sometimes cells loose tissue-specific functions when cultured for an extended time. An alternative to primary cell cultures are immortalized cell lines, which have an extended replicative capacity, a stable phenotype and enable the use of the same consistent cells throughout a research project. In addition, cultivation of immortalized cell lines is simpler and easier to standardize (for overview see [80]). Table 3 summarizes the available cell lines and model systems for an *in vitro* approach of the study of NAFLD.

### 5.7. Co-Culture Model: Interaction of RAW 264.7 Macrophages and AML-12 Cells

**Figure 4:**
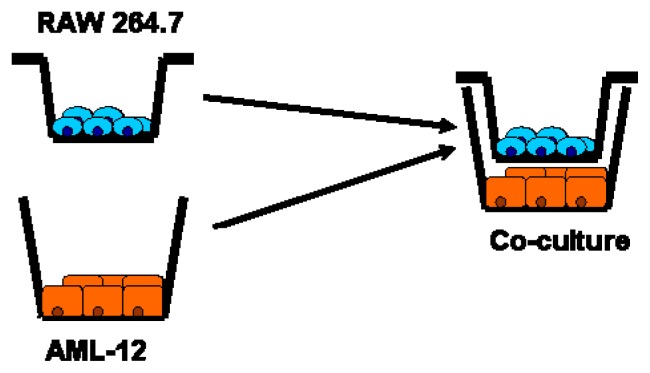
Schematic drawing of co-culture model (as used by Spruss *et al.* [88]).

In the complex architecture of *in vivo* hepatic tissue the interaction of two or more cells is a common phenomenon. Accordingly, reproducing these settings in *in vitro* experiments can be a reliable model to investigate the cell to cell interaction in the progression of disease. For example, a co-culture model was developed by our own group to mimic the effect of feeding a fructose-rich diet on the liver. As it was shown before that chronic intake of fructose is associated with increased portal endotoxin levels suggesting that the effects found on the liver might result from both endotoxin and fructose. RAW 264.7 macrophages and AML-12 cells being models of Kupffer cells and murine hepatocytes, respectively, were concomitantly exposed to fructose and/or LPS in the presence of inhibitors (Figure 4). Indeed, studies have shown that morphological features, phagocytic properties or reactivity to external stimuli (e.g., lipopolysaccharide) was almost similar in both RAW 264.7 macrophages and Kupffer cells [81–84]. Therefore, RAW264.7 cells were used as model cells for Kupffer cells. RAW 264.7 cells were grown on transwell cell culture chambers until 70% confluency. AML-12 cells were grown in normal six-well plates until 70% confluency followed by an 18 h serum starvation. Meanwhile, RAW 264.7 cells were challenged with either 50 ng/mL LPS or fructose either in the presence or in the absence of inhibitors for 1 h. The concentration of LPS used in the *in vitro* experiments is equivalent to 10 EU/mL; however, if these concentrations are comparable to the endotoxin levels found in the portal vein of patients with NAFLD remains to be determined. To our knowledge levels of endotoxin in the portal vein of patients with NAFLD have not yet been determined. Indeed, in most of the human studies endotoxin levels were measured in peripheral blood. Results of our own studies but also those of other groups suggest that in patients with NAFLD/NASH endotoxin levels might vary from 0.3 to 14.8 EU/mL [85–87]. The results obtained from the co-culture model suggest that an activation of Kupffer cells by endotoxin may trigger the induction of inflammatory cytokines in other cell types in the liver (e.g., hepatocytes), which play a vital role in the progression of fructose-induced non-alcoholic fatty liver disease [88].

### 5.8. Three-Dimensional Cell Culture Model

Until now, most of *in vitro* studies have utilized two-dimensional monolayer cell cultures to elucidate the molecular mechanisms involved in the progression of NAFLD; however, recent studies have shown that three-dimensional liver cell cultures may better mimic the physiological settings of the liver rather than two-dimensional monolayer liver cell cultures [89,90]. Indeed, Janokar *et al.* [90] developed a model of 3D spheroids by using elastin-like peptide ELP-polyethyleneimine (PEI) and showed that ELP-PEI coated surfaces influence the morphology of H35 rat hepatoma cells to create 3D spheroids. This model was predominantly used to investigate the transcriptional regulation of genes involved in hepatic steatosis. Furthermore, the results for the first time have shown the transcriptional dynamics (e.g., activation, suppression) of NFκB in response to TNFα stimulation.

Taken together, co-culture models or 3D models which represent the complex architecture of liver tissue might be potential tools to elucidate the cellular mechanisms of the fatty liver disease. Indeed, these models serve as better tools to study cell-to-cell cross talk in the progression of the disease; however, further research is needed to standardize the culture conditions.

## 6. Conclusions

As summarized and discussed in this review, many animal models and herein, particularly rodent models, and to a lesser extend also cell culture models of NAFLD have been developed and been used in recent years to unravel the molecular mechanisms involved in the onset but also the progression of this liver disease; however, the available models, be it *in vivo* or *in vitro* only mimic certain disease aspects found in humans and markedly differ in regards to the degree of hepatocellular damage and metabolic alterations associated with the development of the disease. Nevertheless, when chosen carefully, both *in vitro* and *in vivo* models can be used to verify hypotheses on mechanisms underlying the development of NAFLD and as tools to test new therapeutic and prevention strategies. The future aim should be to develop both animals and cell based models that more closely reflect the histopathology and pathophysiology found in humans with NAFLD, and thereby, increasing the knowledge on the molecular mechanisms involved in the onset but also the progression of NAFLD and providing the basis for the development of better therapeutic approaches to the disease.

## Figures and Tables

**Table 1:** Composition of the Piagen diet [57,58].

Ingredients	g/100 g diet	Ingredients	g/100 g diet
Casein	20.0	Vitamin mixture	1
DL methionine	0.3	Choline bitartrate	0.2
Corn strach	15.0	Corn oil	5
Sucrose	48.7	Sodium cholate	0.3
Cellulose powder	5	Cholesterol	1
Mineral mixture	3.5		

**Table 2:** Composition of a liquid based control and Western style diet.

Control diet	% of energy from Nutrients	Western style diet	% of energy from Nutrients
Total sugars	23.3	Fructose	50
Starch	39	Glucose	5
Protein	11	Starch	5
Fat	24	Protein	15
		Cholesterol	0.2
		Fat	25

**Table 3:** Summary of available cell lines and cell culture models [80].

*In vitro* models	Cell lines	Pros	Cons
Primary cell cultures	Hepatocytes from NAFLD patients/rodents Kupffer cells/stellate cells/iNKT cells from human patients/rodents	Mimic *in vivo* settings	Isolation problems Ethical issues Varying reproducibility in experiments Limited culture time
Immortalized cell lines	RAW 264.7, AML-12, J774A, HepG2, HuH7, H4IIE, H4IIEC3, PAV-1, LX2	Continuous growth Easy to culture Stable phenotype	Expression of several enzymes and nuclear factors alter according to the immortalization method
Co-culture models	RAW 264.7 and AML-12 Human hepatocytes and adipocytes	Mimic *in vivo* liver architecture Important tools in cellular cross talk studies	Difficult to cultivate
3D cultures	H35 rat hepatoma cell line	Mimic *in vivo* liver architecture Liver specific differentiation and function Tools for transcriptional regulation studies	Difficult to cultivate

## Abbreviations

NAFLD: non-alcoholic fatty liver disease
NFκB: nuclear factor kappa B
ROS: reactive oxygen species
TNFα: tumor necrosis factor α
TG: triglycerides
IL-6: interleukin 6
MCD: methionine- and choline-deficient
MC4R: melanocortin 4 receptor
HFD: high-fat diet
SREBP1: sterol regulator element-binding protein 1c
HFCS: high fructose corn syrup
